# Interaction of PIAS1 with PRRS virus nucleocapsid protein mediates NF-κB activation and triggers proinflammatory mediators during viral infection

**DOI:** 10.1038/s41598-019-47495-9

**Published:** 2019-07-30

**Authors:** Hanzhong Ke, Sera Lee, Jineui Kim, Hsiao-Ching Liu, Dongwan Yoo

**Affiliations:** 1 Department of Pathobiology, University of Illinois at Urbana-Champaign, Urbana, IL USA; 2 Department of Animal Science, North Carolina State University, Raleigh, NC USA

**Keywords:** Viral infection, Viral pathogenesis

## Abstract

Porcine reproductive and respiratory syndrome virus (PRRSV) activates NF-κB during infection. We examined the ability of all 22 PRRSV genes for NF-κB regulation and determined the nucleocapsid (N) protein as the NF-κB activator. Protein inhibitor of activated STAT1 (signal transducer and activator of transcription 1) (PIAS1) was identified as a cellular protein binding to N. PIAS1 is known to bind to p65 (RelA) in the nucleus and blocks its DNA binding, thus functions as a repressor of NF-κB. Binding of N to PIAS1 released p65 for NF-κB activation. The N-terminal half of PIAS1 was mapped as the N-binding domain, and this region overlapped its p65-binding domain. For N, the region between 37 and 72 aa was identified as the binding domain to PIAS1, and this domain alone was able to activate NF-κB. A nuclear localization signal (NLS) knock-out mutant N did not activate NF-κB, and this is mostly likely due to the lack of its interaction with PIAS1 in the nucleus, demonstrating the positive correlation between the binding of N to PIAS1 and the NF-κB activation. Our study reveals a role of N in the nucleus for NF-κB activation and proinflammatory cytokine production during infection.

## Introduction

Many viruses utilize NF-κB signaling pathway for their own benefit^1–3^. Porcine reproductive and respiratory syndrome virus (PRRSV) is a single-stranded positive-sense RNA virus that infects domestic and wild pigs and causes PRRS which is one of the most economically important diseases in the swine industry worldwide^4,5^. The disease is characterized by severe respiratory stress in nursing pigs and reproductive failure in sows and gilts^5^. PRRSV primarily infects pulmonary alveolar macrophages (PAMs) and dendritic cells (DCs) in the tonsils, upper respiratory tract, and lungs^6,7^. PRRSV infection of pigs induces inflammation in the lungs, which is characterized by the increase of proinflammatory cytokines including IL-1β, IL-6, IL-8, and TNF-α and by the recruitment of monocytes and neutrophils to affected sites^8–11^. The recruitment of fresh neutrophils and monocytes to affected sites provides more target cells for PRRSV to replicate and contributes to pathogenesis^10,12^. Since proinflammatory cytokines are elevated^13–16^, and NF-κB signaling is activated in PRRSV-infected cells^17–21^, it seems evident that PRRSV activates NF-κB and causes the NF-κB-mediated production of proinflammatory cytokines, which is correlated with PRRSV pathogenesis. Despite the potential role of NF-κB for viral pathogenesis, the underlying mechanism for NF-κB activation by PRRSV remains unknown.

NF-κB is a family of transcription factors consisting of RelA (p65), RelB, NF-κB1 (p50 and its precursor p105), NF-κB2 (p52 and its precursor p100), and c-Rel for homo/heterodimers with RelA or RelB^22^. NF-κB can be activated by proinflammatory cytokines such as tumor necrosis factor-α (TNF-α) and interleukin-1 (IL-1). Upon stimulation, NF-κB becomes phosphorylated and translocated to the nucleus where it binds to κB sites, which are the specific DNA loci for NF-κB. NF-κB regulates the expression of a variety of proinflammatory cytokine genes including IL-1β, IL-6, IL-8, and TNF-α^23^ and anti-inflammatory cytokines such as IL-10^24^. The proinflammatory cytokines will further activate NF-κB signaling in the autocrine manner^25^. Dysregulation of NF-κB may result in disease, and thus maintaining the homeostasis of NF-κB is crucial for the health of a host^22,26,27^. To maintain NF-κB homeostasis from over activation, negative feedback mechanisms are available. IκB is a negative regulator of NF-κB in the cytoplasm. By ubiquitination and degradation of IκB, NF-κB is released and activated for NF-κB-regulated gene expression. In the nucleus, PIAS1 [protein inhibitor of activated STAT (signal transducer and activator of transcription)] functions as a negative regulator of NF-κB. By specific binding to p65, PIAS1 prevents the NF-κB dimer from docking to κB sites on DNA such that NF-κB-mediated gene expression is tightly controlled. This action occurs in the nucleus since PIAS1 predominantly resides in the nucleus^28^.

PIAS1 is one of four members of the PIAS family including PIASx (also known as PIAS2), PIAS3, and PIASy (also known as PIAS4)^29,30^. Except PIAS1, which is the sole form, three other PIAS isoforms are expressed via mRNA splicing. The PIAS genes are expressed ubiquitously in mammals, and their homologs are also found in *Drosophila* as dPIAS/Zimp^31,32^ and in yeast as SIZ1 and SIZ2^33^, suggesting the broad existence of PIAS proteins in the eukaryotic kingdom. The PIAS family proteins share a high sequence identity of more than 40% attributed to several conserved functional domains^34^. Among the four member proteins, PIAS1 is the largest. PIAS1 contains Scaffold-attachment factor (SAF)-A and SAF-B, Apoptotic chromatin-condensation inducer in the nucleus (ACINUS), and PIAS domain (SAP) in its N-terminal region, a Pro-Ile-Asn-Ile-Thr (PINIT) motif and a CCCHCCCC-motif-type RING-finger-like zinc-binding domain (RLD) in the middle region, and a highly acidic domain (AD) and a serine and threonine rich (S/T) in the C-terminal region^35^. These motifs are highly conserved and shared in the PIAS family. The PIAS proteins were initially identified as negative regulators of STAT signaling^36,37^. Subsequently, PIAS1 has been shown to contain a SUMO-E3 ligase activity and regulates p53 tumor suppressor through this activity^38^. PIAS1 has also been shown to negatively regulate the interferon-stimulated genes (ISGs) of innate immunity^39^ and the NF-κB signaling^28^.

PRRSV has been investigated for NF-κB activation to a certain extent but the molecular basis of action is unknown^20,40,41^. The nucleocapsid (N) protein of PRRSV is a small protein of 128 and 123 amino acids for genotypes I and II, respectively, and is the most abundant viral protein expressed during infection^42^. N is localized in the cytoplasm of infected cells but is also specifically distributed in the nucleus and nucleolus^42,43^. N contains a nuclear localization signal (NLS), and its nuclear localization is NLS-dependent through binding of N to importin-α and importin-β^44^. Even though the exact function of N in the nucleus is still unclear, its clinical role has been studied in pigs using NLS-null mutant PRRSV. The NLS-null virus-infected pigs show clinical attenuation and short duration of viremia as well as high titers of neutralizing antibodies^45,46^. Such studies demonstrate that N protein nuclear localization plays an important role for pathogenesis during infection. Yeast two-hybrid assays using the cDNA libraries from MARC-145 cells and PAMs have identified multiple cellular proteins interacting with N, which includes fibrillarin^47^, I-mfa domain containing protein (HIC)^48^, nucleolin, B23, poly-A binding protein^49,50^, importin-α, importin-β, and exportin^44,51^. PIAS1 has also been identified as a molecular partner of N in our laboratory (unpublished data) and by two others^50^ (personal comunication with H.C. Liu, North Carolina State University, Raleigh, NC).

The identification of the N-PIAS1 interaction by three independent laboratories has led us to study the biological consequence of the binding of N to PIAS1. Since PIAS1 can function as a repressor for NF-κB^28^, we hypothesize that PRRSV N may neutralize this function by binding to PIAS1. In the present study, we show that PRRSV N binds to the N-terminal half of PIAS1 which overlaps the p65 binding domain. N does not bind to p65, and thus the binding of PIAS1 to N and p65 is competitive, which results in the release of p65 from PIAS1 by N binding, leading to activation of NF-κB. The binding domain of N to PIAS1 overlaps NLS, which reveals a novel function of N in the nucleus for NF-κB activation and induction of proinflammatory cytokines during infection.

## Results

### NF-κB activation in PRRSV-infected cells

**Figure 1:**
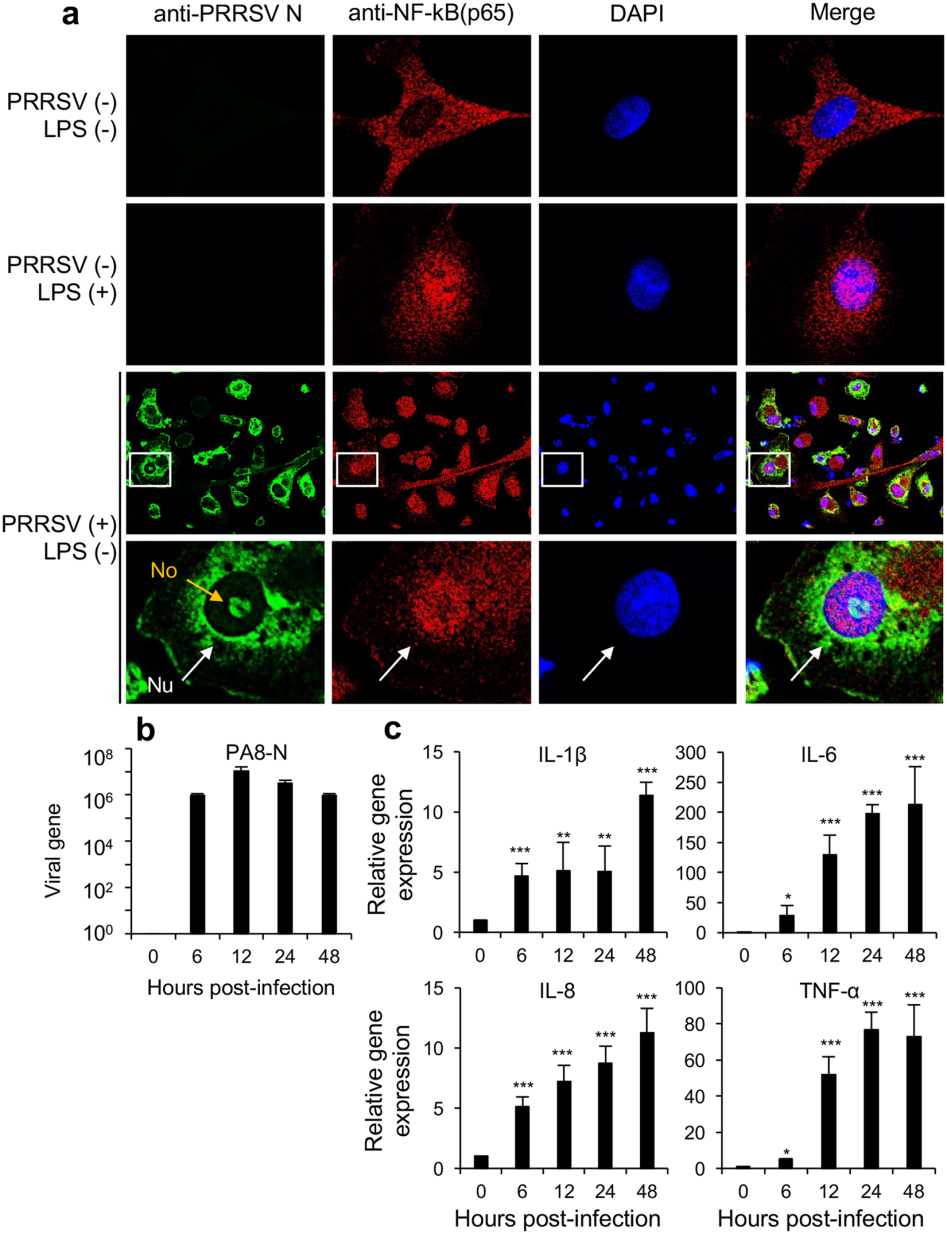
Activation of NF-κB by PRRSV in ZMAC cells and primary porcine alveolar macrophages (PAMs). (**a**) ZMAC cells were infected with the PA8 strain of PRRSV at 5 moi for 10 h. Cells were stained with α-PRRSV-N MAb (MR40) (mouse) (green) and α-p65 PAb (rabbit) (red). The nuclei were stained with DAPI (blue). Images were taken by confocal microscopy (Nikon A1R). White arrows indicate the nucleus (Nu), and the orange arrow indicates the nucleolus (No). Scales in micrometers (μm). (**b**,**c**) Primary PAMs were infected with PRRSV at 1 moi for indicated times, and total RNAs were isolated. RT-qPCR was performed to detect the levels of PRRSV-N genes (**b**) and transcripts for IL-1β, IL-6, IL-8, and TNF-α (**c**). The relative levels were calculated using the 2^−ΔΔCT^ method by normalizing the values to that of β-actin. The fold changes were compared to the levels at time zero. Error bars, mean ± standard deviation (s.d.). (n = 3). *P < 0.05, **P < 0.01, ***P < 0.001.

To examine the regulation of NF-kB during infection, we infected ZMAC cells with PRRSV at 5 multiplicity of infection (moi) for 10 h. ZMAC is an established cell line of porcine alveolar macrophages, which are natural target cells for PRRSV, derived from lungs of porcine fetus^52^. The cells were stained with p65-specific polyclonal antibody (PAb) and PRRSV N-specific monoclonal antibody (MAb) MR40. As anticipated, p65 was distributed in the cytoplasm of ZAMC cells when not stimulated (Fig. 1a). When stimulated by adding lipopolysaccharide (LPS; 100 ng/ml) however, p65 predominantly accumulated in the nucleus (Fig. 1a), indicating the activation of NF-κB in the macrophages. In PRRSV-infected cells, almost all of PRRSV-N positive ZMAC cells exhibited p65 accumulation in the nucleus even without LPS treatment (Fig. 1a), demonstrating the activation of NF-κB by PRRSV.

Activation of NF-κB triggers expression of proinflammatory cytokines^23^. Thus, we examined the expression of IL-1β, IL-6, IL-8, and TNF-α in primary porcine alveolar macrophages (PAMs) during infection. Cells were infected with PRRSV at 1 moi for up to 48 h, and quantitative RT-PCR was conducted to measure respective transcripts. The kinetics of PRRSV replication was determined using the levels of N gene expression in virus-infected cells (Fig. 1b). The N gene expression reached the peak at 12 h pi followed by gradual decrease until 48 h pi. The mRNA for IL-1β significantly (*P* = 0.00043) increased as early as 6 h postinfection (pi). Thereafter, IL-1β expression remained consistent until 24 h pi and reached its peak at 48 h pi. The elevated expression of IL-1β confirmed the activation of NF-κB in PAMs by PRRSV. The kinetics of IL-1β expression suggests the NF-κB activation was gradual and accumulating. Similar patterns of induction were observed for IL-6, IL-8, and TNF-α (Fig. 1c). Together, these data demonstrate the activation of NF-κB by PRRSV.

### Identification of NF-κB activating proteins of PRRSV

**Figure 2:**
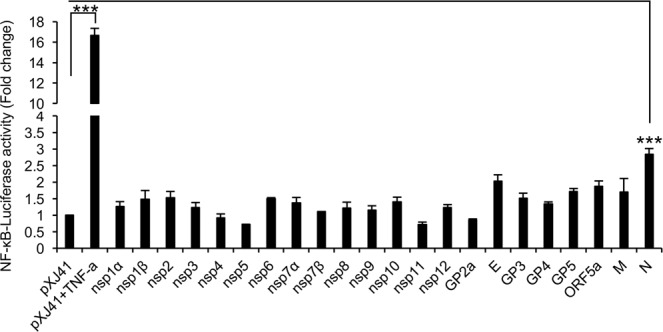
Identification of NF-κB activating proteins of PRRSV. NF-κB luciferase activities of nonstructural proteins and structural proteins. HeLa cells grown in 12-well plates were transfected with pNF-κB-Luciferase (0.5 μg), pRL-TK (0.05 μg), and each (0.5 μg) of indicated PRRSV genes for 24 h. Cells were treated or mock-treated with TNF-α (20 ng/ml) for 6 h, and cell lysates were prepared in passive lysis buffer for luciferase assays as described in Materials and Methods. Relative luciferase activities were obtained by normalizing the firefly luciferase to *Renilla* luciferase activities. Values of the relative luciferase activity in the pXJ41 control group were set as 1 and the values for individual viral proteins were normalized using that of the pXJ41 control. Error bars, mean ± standard deviation (s.d.). (n = 3). ***P < 0.001.

The roles that PRRSV plays for modulation of NF-κB signaling are complicated. PRRSV activates NF-κB and stimulates proinflammatory cytokine production in virus-infected macrophages and pigs^8–21,40^. (Fig. 1). In other studies however, nonstructural protein (nsp) 1α, nsp1β, nsp4, and nsp11 suppress NF-κB signaling^53–58^, whereas nsp2 activates^59^ or inhibits^60^ NF-κB depending on the strains of PRRSV. To identify viral proteins regulating NF-κB signaling, all 22 coding sequences of PRRSV except nsp2TF and nsp2N were individually cloned with a FLAG tag and expressed in HeLa cells as a fusion protein. Then, 14 nsps and 8 structural proteins were individually examined for their ability to modulate NF-κB in HeLa cells by the promoter-based reporter assay (Fig. 2). The relative luciferase activities were obtained by normalizing the firefly luciferase to *Renilla* luciferase activities. The value of the relative activity in the empty vector (pXJ41) control group was set as 1 without TNF-α treatment. To compensate the variations of reporter activities, we took 2-fold induction as the cutoff for significance activation. The NF-κB activity increased slightly by 1.5-fold without TNF-α stimulation in nsp2-expressing cells, and this was the only protein activating NF-κB among all 14 nsps. However, this value was below the 2-fold cutoff and thus was considered insignificant, and we concluded that no nsp activated NF-κB signaling. For structural proteins, the E protein and the nucleocapsid (N) protein induced NF-κB signaling by more than 2-fold (2.01 and 2.83-fold, respectively), indicating the E and N proteins were NF-κB activators, with N being more potent than E. The other structural proteins GP5, ORF5a, and M showed a tendency towards inducing NF-κB signaling, but their induction levels were lower than 2-fold, and we concluded their abilities to induce NF-κB signaling were negligible.

### PRRSV nucleocapsid (N) protein-mediated NF-κB activation

**Figure 3:**
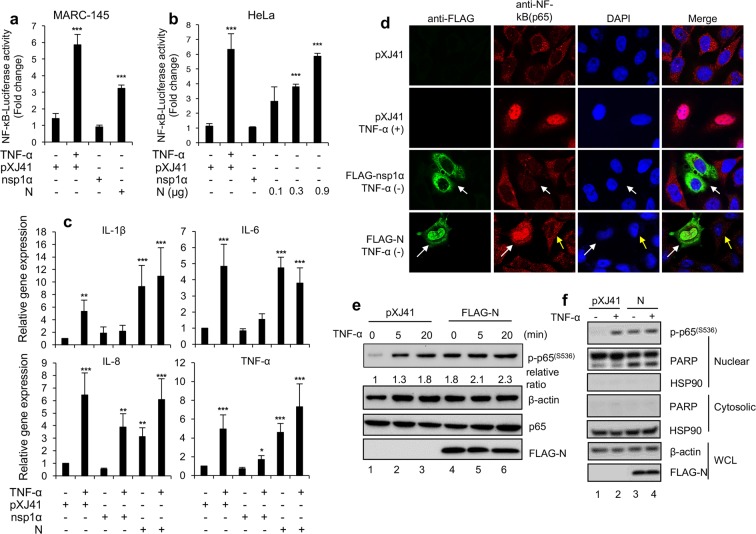
NF-κB activation by N. (**a**,**b**) Cells were co-transfected with pNF-κB-Luciferase (0.5 μg) and pRL-TK (0.05 μg), along with 0.5 μg indicated genes. After 24 h (HeLa cells) or 42 h (MARC-145 cells), cells were treated or mock-treated with TNF-α (20 ng/ml) for 6 h. Luciferase reporter activities were calculated by normalizing to the *Renilla* activity. Error bars, mean ± s.d. (n = 3). Significance was calculated by comparing to pXJ41. ***P < 0.001. (**c**) MARC-145 cells were transfected with 2 μg of indicated genes for 42 h and treated with or mock-treated with TNF-α (20 ng/ml) for 6 h. The expression levels were calculated with 2^−ΔΔCT^ method by normalizing to that of β-actin. The fold changes were calculated with respect to the level of pXJ41. Error bars, mean ± s.d. (n = 3). *P < 0.05, **P < 0.01, ***P < 0.001. (**d**) HeLa cells were transfected with 2 μg of indicated genes for 24 h and treated or mock-treated with TNF-α (20 ng/ml) for 20 min. The cells were stained with indicated antibodies. Nuclei (blue) were stained with DAPI. White arrows indicate nsp1α or N-expressing cells. (**e**,**f**) HeLa cells were transfected with 2 μg of indicated genes for 30 h and stimulated with TNF-α (20 ng/ml) for indicated times (**e**) or 20 min (**f**). Cells lysates (**e**) or cytoplasmic and nuclear fractions (**f**) were prepared and subjected to Western blot. HSP90 and PARP serve as indicators for the cytoplasmic and nuclear fractions, respectively. Intensities of phosphor-p65(S536) staining were quantified with the FluroChem R system, normalized to that of β-actin, and compared to the pXJ41 control (TNF-α mock-treated) (Lane 1) (**e**). Numbers below each band in the top panel indicate relative fold changes.

Since N was demonstrated as the NF-κB activator in HeLa cells (Fig. 2), this activity was further studied in other cell types. MARC-145 cells are permissive for PRRSV^61^ and they were transfected with the NF-κB luciferase reporter and pRL-TK *Renilla* control plasmids along with an empty vector (pXJ41), nsp1α gene, or N gene for 42 h. Cells were then treated with TNF-α for 6 h, and the reporter expression was examined (Fig. 3a). Compared to mock-treated pXJ41 group, TNF-α treatment stimulated the luciferase activity by 4-fold in MARC-145 cells, indicating the normal activation of NF-κB signaling in the assay system. PRRSV nsp1α was known to suppress NF-κB signaling^55,56^ and thus, no activation of NF-κB was observed by nsp1α as expected (Fig. 3a). In contrast, N activated NF-κB signaling significantly (*P* = 0.0000821) although it was not as much as TNF-α stimulation (Fig. 3a), indicating the N-mediated NF-κB activation was not due to different cell types. The N-mediated NF-κB activation was determined using different amounts of N, and the signaling activation was positively correlated with the increasing amounts of N (Fig. 3b), indicating the dose-dependent activation of NF-κB by N.

We next examined the expression of proinflammatory cytokines mediated by N. MARC-145 cells were transfected with N gene for 42 h and treated with TNF-α for 6 h. Specific transcripts were then determined by RT-qPCR for IL-1β, IL-6, IL-8, and TNF-α (Fig. 3c). The cytokine expressions were upregulated by N even without TNF-α stimulation, while pXJ41 or nsp1α as a negative control did not upregulate these cytokines. When stimulated with TNF-α, nsp1α suppressed these genes whereas pXJ41 and N elevated the expression of these cytokines. Together, these data showed that the N protein activates NF-κB signaling to promote the production of proinflammatory cytokines. NF-κB activation is mediated through the phosphorylation of p65 at position Ser536 followed by the translocation of phosphorylated p65 to the nucleus^22^. Thus, it was of interest to examine the subcellular distribution of p65 in cells expressing N using anti-p65 PAb and anti-FLAG MAb. Without stimulation, p65 was distributed in the cytoplasm of cells, but upon stimulation with TNF-α, it was translocated to the nucleus (Fig. 3d, panels in second row). In nsp1α-expressing cells, p65 was distributed in the cytoplasm despite the stimulation (Fig. 3d, panels in third row), indicating the suppression of p65 activation by nsp1α^55,56^. In N-expressing cells however, p65 accumulated predominately in the nucleus (Fig. 3d, panels in fourth row), indicating the p65 activation by N. The phosphorylation of p65 is an additional feature of activated NF-κB, and thus was also examined in N protein-expressing cells using a phosphorylated p65 (p-p65^S536^)-specific antibody (Fig. 3e). The basal level of phosphorylated p65 (p-p65^S536^) in HeLa cells increased rapidly when treated with TNF-α. In cells expressing N protein however, p-p65^S536^ increased by two-fold even without TNF-α stimulation comparing to that of the basal level. The treatment of TNF-α for 5 min or 20 min enhanced p-p65^S536^ in N-expressing cells, showing that the synergistic phosphorylation of p65 by TNF-α and N. Since N induced p65 phosphorylation, its nuclear translocation was examined by Western blot using p-p65^S536^ antibody of the nuclear and cytoplasmic fractions from the cells expressing N (Fig. 3f). The p-p65^S536^ band was meager in the nuclear fraction of the control cells (lane 1) but became increased when stimulated with TNF-α (lane 2). In N-expressing cells, p-p65^S536^ accumulated in the nuclear fraction even without TNF-α treatment (lane 3) compared to control, demonstrating the phosphorylation of p65 by N and the nuclear translocation of phosphorylated p65.

### Binding of PRRSV nucleocapsid (N) protein to PIAS1

**Figure 4:**
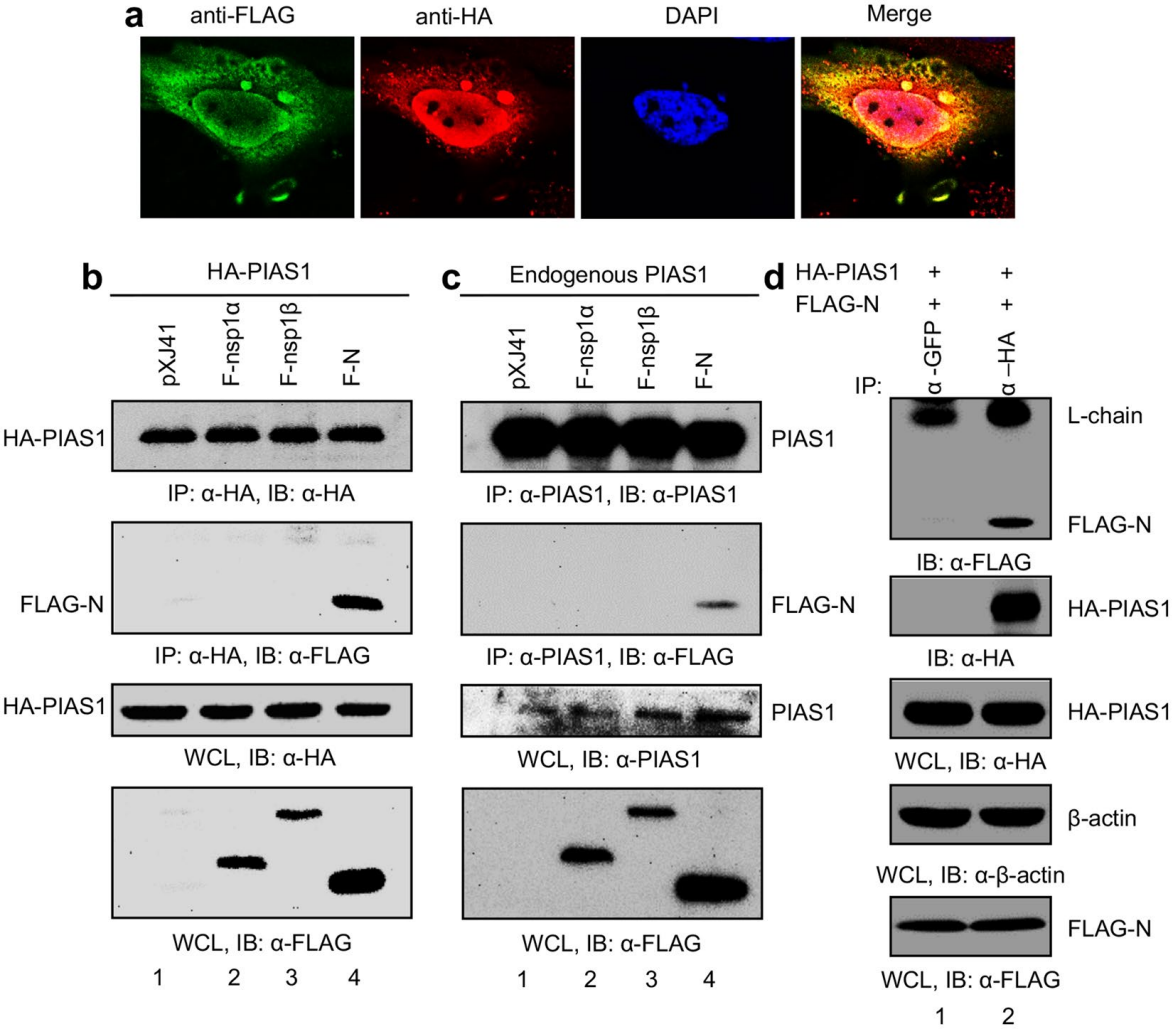
Identification of PIAS1 as a molecular partner of N. (**a**) Colocalization of N and PIAS1. HeLa cells were transfected with FLAG-N for 24 h and stained with α-FLAG MAb (green) and α-PIAS1 PAb (red). Nuclei (blue) were stained with DAPI. (**b**,**c**) Specific binding of N with exogenously expressing PIAS1 (**b**) and endogenous PIAS1 (**c**) determined by co-IP. HeLa cells were co-transfected with 1 μg of pXJ41, F-nsp1α, F-nsp1β, or N plasmids, along with HA-PIAS1 (2 μg) for 30 h, and were subjected to co-IP. Cells lysates were pulled down by α-HA MAb and were probed with α-FLAG PAb (middle panel) or α-HA MAb (top panel). Whole cell lysates (WCL) were resolved and probed with α-HA MAb (third panel) or α-FLAG PAb (bottom panel). (**d**) HeLa cells were co-transfected with the HA-PIAS1 (2 μg) and FLAG-N (1 μg) plasmids for 30 h and subjected to co-IP. Cell lysates were pulled down with α-GFP MAb or α-HA MAb followed by probing with α-FLAG MAb (top panel) or α-HA MAb (second panel). WCL were probed with α-HA MAb (thrid panel) or α-FLAG MAb (bottom panel). β-actin served as a loading control.

The basis for NF-κB activation by N remains unknown, and we attempted to identify cellular proteins interacting with N by yeast two-hybrid screening using N as the bait. The screening results showed that poly-A binding protein (PABP), human inhibitor of MyoD family a (I-mfa) domain containing protein (HIC), and protein inhibitor of activated STAT1 (PIAS1) were among the molecular partners of N. Of these proteins, N interactions with PABP and HIC have been reported^48,50^, and PIAS1 was of our particular interest. PIAS1 has been reported as a negative regulator of NF-κB^28^, and so we hypothesized that N protein neutralized the PIAS1 activity by binding to it. To test this hypothesis, we first examined the colocalization of N and PIAS1 by IFA. HeLa cells were co-transfected with the FLAG-N and HA-PIAS1 plasmids, and IFA was conducted using anti-FLAG MAb and anti-HA MAb. The N protein was distributed in the cytoplasm and nucleus as anticipated (Fig. 4a). PIAS1 is reported to distribute in the cytoskeleton, cytoplasm, and majorly nucleus^28,62^, and in the N and PIAS1 co-expressing cells, both proteins were found to colocalize in the cytoskeleton, cytoplasm, and nucleus (Fig. 4a), suggesting the binding of N to PIAS1. We confirmed their binding by performing a co-immunoprecipitation (co-IP) assay. Cells were co-transfected with the HA- PIAS1 and FLAG-N plasmids, and cell lysates were incubated with either anti-HA MAb or anti-PIAS1 MAb, followed by immunoblot using anti-FLAG Ab. Regardless of whether the PIAS1 expression was exogenous (Fig. 4b) or endogenous (Fig. 4c), N was pulled down by PIAS1 (second panel from top, lane 4), whereas FLAG-tagged nsp1α (F-nsp1α) or FLAG-tagged nsp1β (F-nsp1β) was not precipitated by PIAS1. To rule out a possibility of non-specific binding due to the overexpression of N or non-specific reactivity of the Abs, we performed additional co-IP for which cells were co-transfected with an equal amount of the HA-PIAS1 and FLAG-N plasmids, followed by pull-down assays using GFP MAb or HA MAb and immunoblotting with FLAG PAb (Fig. 4d). FLAG-N was again coprecipitated by HA MAb (lane 2) while it was not coprecipitated by GFP MAb (lane 1), confirming the specific interaction of FLAG-N and PIAS1.

### Competition binding of N to PIAS1 and release of p65

**Figure 5:**
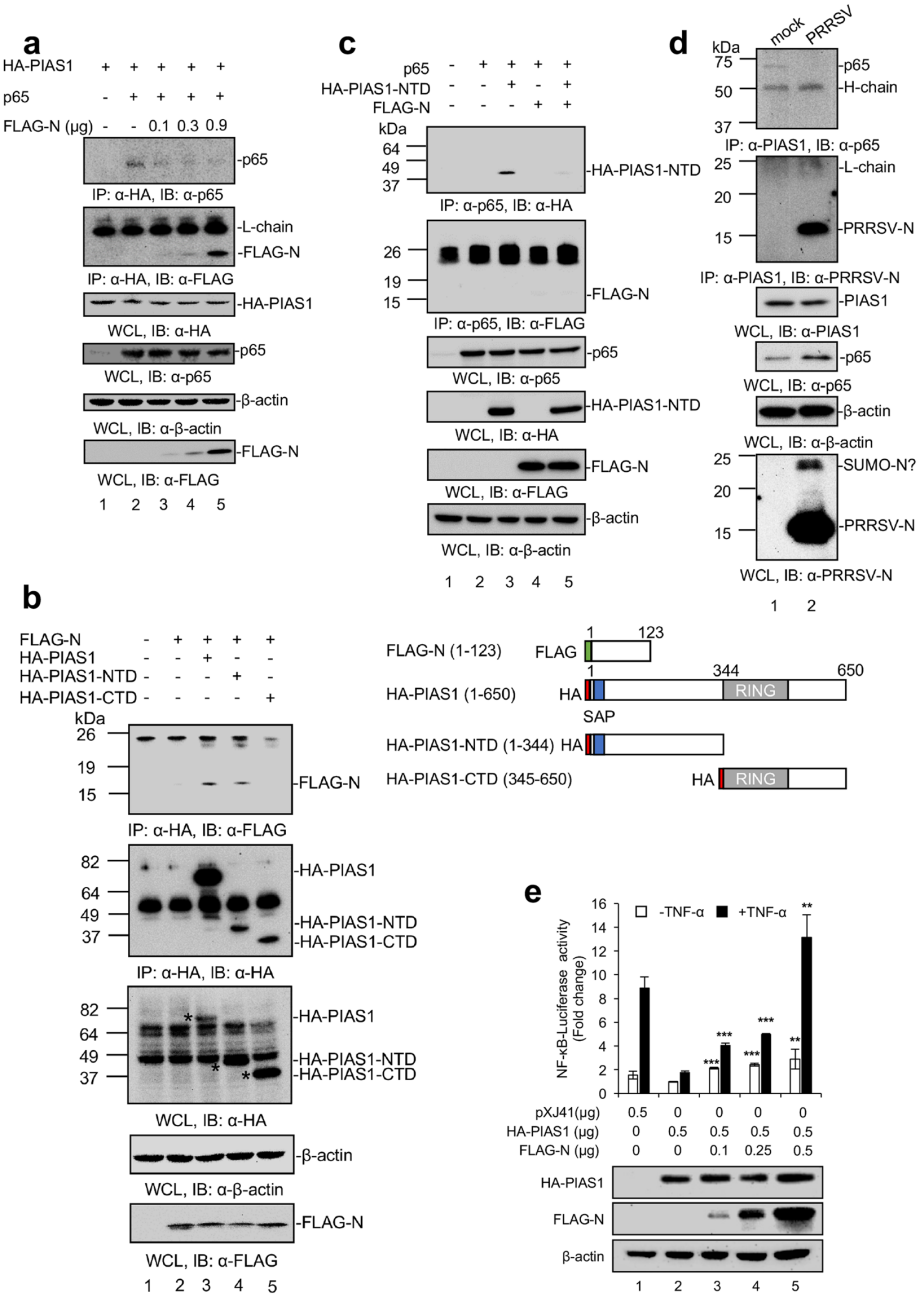
Competition binding of N and p65 to PIAS1. (**a**) HeLa cells were transfected with the HA-PIAS1 and p65 plasmids along with increasing amounts of N gene for 30 h, and cell lysates were subjected to co-IP using α-HA MAb for precipitation and indicated antibodies for immunoblot. (**b**) Schematic presentation of FLAG-tagged N or HA-tagged full length PIAS1 and construction of HA-PIAS1-NTD (N-terminal domain) and HA-PIAS1-CTD (C-terminal domain). Lower panels, co-IP experiments in which HeLa cells were transfected with N plasmid along with HA-PIAS1, HA-PIAS1-NTD, or HA-PIAS1-CTD plasmids as indicated for 30 h, and cell lysates were subjected to co-IP using α-HA MAb for precipitation and using indicated antibodies for immunoblot. Asterisks (*) indicate the HA-specific bands. (**c**) HeLa cells were transfected with p65 along with HA-PIAS1-NTD or FLAG-N or both for 30 h, and cell lysates were subjected to co-IP using α-p65 MAb for precipitation and indicated antibodies for immunoblot. (**d**) Approximately 2 million cells of MARC-145 were infected with PRRSV at 5 moi for 48 h. Cell lysates were prepared and subjected to co-IP using α-PIAS1 MAb and immunoblot using α-p65 MAb or α-PRRSV-N PAb (bottom panel). β-actin served as a loading control. (**e**) N expression rescued NF-kB activity from PIAS1-mediated suppression. HeLa were transfected with NF-kB-luciferase reporter (0.5 μg) and pRL-TK plasmid (0.05 μg) along with PIAS1 plasmid (0.5 μg) alone or with PIAS1 (0.5 μg) and variable amounts of N plasmids together. Protein expressions were determined by Western blot using α-HA MAb, α-FLAG PAb, and α-β-actin MAb. β-actin served as a loading control. 24 h post-transfection, cells were stimulated with TNF-α (20 ng/ml) for 6 h. Error bars, mean ± s.d. (n = 3). Statistical significance was calculated by comparing the values to PIAS1 alone. *P* values were calculated using two-tailed Student’s *t*-test. **P < 0.01, ***P < 0.001.

Since PIAS1 is known to bind to p65 of NF-κB and inhibits p65 binding to DNA^28^, we anticipated that the N-PIAS1 binding would disrupt the PIAS1-p65 interaction, which would result in the release of PIAS1 from p65 and the activation of NF-κB. To test this hypothesis, we conducted a competition co-IP assay. Cells were transfected with the HA-PIAS1 and p65 plasmids along with increasing amounts of N gene (Fig. 5a). Lysates were incubated with anti-HA Mab, and the precipitates were subjected to Western blot using p65 MAb or FLAG PAb. p65 was pulled down by PIAS1 when N was absent (top panel, lane 2), confirming the PIAS1-p65 binding. When N was expressed however, the amount of p65 decreased (top panel), and the decrease of p65 was proportional to the increasing amounts of N (second panel). This finding indicates the competition between N and p65 for binding to PIAS1, and thus N-binding to PIAS1 may result in the release of p65 from PIAS1.

Since the N-terminal 344 amino acids of PIAS1 contains the p65 binding region^28^, and because N competes with p65 for PIAS1 binding, we wanted to determine if the p65 binding region in PIAS1 was also responsible for the N-binding. Two truncations were made to represent 1–344 aa and 345–650 aa of PIAS1 and they were designated HA-PIAS1-NTD (N-terminal domain) and HA-PIAS1-CTD (C-terminal domain), respectively (Fig. 5b). We then performed co-IP experiments using cells over-expressing PIAS1 truncations and N. The N protein was precipitated by HA-PIAS1 (lane 3) and by HA-PIAS1-NTD (lane 4), whereas it was not precipitated by HA-PIAS1-CTD (lane 5). This result demonstrated the N-terminal 344 amino acids of PIAS1 was the N protein-binding region. This is the same region as the p65-binding region of PIAS1 and further supports that N and p65 compete from each other to bind to PIAS1.

If N bound to p65, it would also result in the disruption of p65-PIAS1 interaction, and thus, we examined if N was also able to bind to p65. We conducted co-IP using cells over-expressing p65 and N (Fig. 5c). Cell lysates co-expressing HA-PIAS1-NTD and N were incubated with p65 MAb, and the precipitates were subjected to Western blot with HA MAb or FLAG PAb. For this experiment, HA-PIAS1-NTD was preferred to full-length HA-PIAS1 since the expression level of HA-PIAS1-NTD was higher than that of HA-PIAS1 (Fig. 5b, third panel), and so it would be harder for N to block the HA-PIAS1-NTD binding to p65. HA-PIAS1-NTD was pulled down by p65 (Fig. 5c, top panel, lane 3), and the amount of HA-PIAS1-NTD was significantly reduced when N protein was present (lane 5). This further confirms that PIAS1-NTD binding to p65 is inhibited by N. Notably, N did not bind to p65 (Fig. 5c, second panel, lanes 4 and 5). These results demonstrated that the disruption of PIAS1-p65 interaction was not due to the binding of N to p65 but due to the binding of N to PIAS1-NTD.

The competition between PIAS1 and N for p65 binding was also examined in virus-infected cells. MARC-145 cells were infected with PRRSV at 5 moi for 48 h, and endogenous PIAS1 was precipitated using MAb from the cell lysates, and the precipitates were resolved and probed with p65 MAb and N PAb. It was evident that p65 was pulled down by PIAS1 MAb in mock-infected cells (Fig. 5d, top panel, lane 1). In virus-infected cells however, p65 was not pulled down, and instead, N was pulled down by PIAS1 (Fig. 5d, second panel, lane 2), confirming the competition between N and PIAS1 for p65 binding in PRRSV-infected cells.

To determine the functional consequence of N-PIAS1 interaction, the NF-κB luciferase assay was performed in cells expressing PIAS1 or N (Fig. 5e). Cells transfected with an empty vector elicited 10-fold induction of NF-κB activity when stimulated with TNF-α. In contrast, the NF-κB activity decreased dramatically when PIAS1 was over-expressed, indicating the suppression of NF-κB by PIAS1. When the N expression was increased (Fig. 5e, bottom Western-blot bands), however, the NF-κB activity was reversed. Together, these data confirm that N binding to PIAS1 prevents PIAS1 binding to p65, thereby frees up p65 for activation.

### Identification of PIAS1 binding region in N

**Figure 6:**
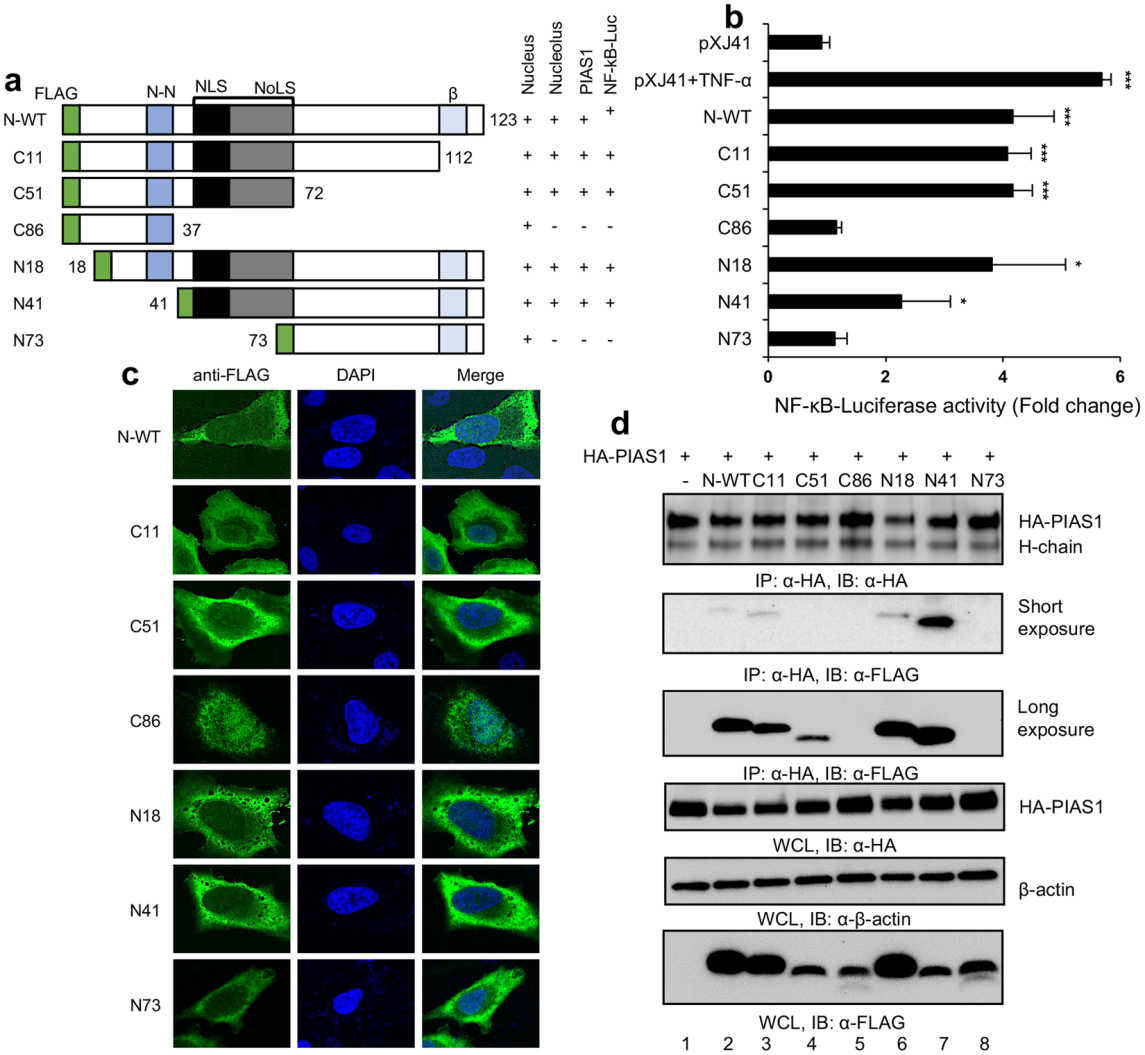
NF-κB binding domain of PIAS1 functions for N binding. (**a**) Schematic presentation of N and construction of N mutants. NLS, nuclear localization signal; N-N, non-covalent-interaction domain for dimerization of N; NoLS, nucleolar localization signal; β, β-sheet responsible for overall conformation of N. Numbers indicate amino acids positions. N-FL, full length N. Nuclear and nucleolar localizations, PIAS1 binding, and NF-κB activation of N and its mutants are summarized as positive (+) and negative (−). (**b**) NF-κB activities of N mutants. HeLa cells were transfected with plasmids containing the plasmids for NF-κB-Luciferase (0.5 μg), pRL-TK (0.05 μg), and individual mutants of N (0.5 μg) for 24 h. Cells were treated or mock-treated with TNF-α (20 ng/ml) for 6 h. Cell lysates were prepared and subjected to luciferase assays as described in Materials and Methods. Relative luciferase activities were obtained by normalizing the firefly luciferase to *Renilla* luciferase activities. Error bars, mean ± s.d. (n = 3). Significance was calculated by comparing to pXJ41 using two-tailed Student’s *t*-test. *P < 0.05, **P < 0.01, ***P < 0.001. (**c**) Cellular localization of N mutants. HeLa cells were transfected with plasmids containing the N gene or each of its mutants (2 μg) of which a FLAG tag was fused at the N-terminus of each construct. At 24 h post-transfection, cells were stained with α-FLAG MAb (green) to indicate N distribution. Nuclei were stained with DAPI (blue). (**d**) Co-IP for HA-PIAS1 and N mutants according to description for Fig. 4b.

PRRSV N contains multiple motifs; nuclear localization signal (NLS)^43,44^, a noncovalent N-N interaction domain^63^, a nucleolar localization signal (NoLS)^43,51^, phosphorylation sites, and a β-sheet domain^64^ (Fig. 6a). To determine the domain in N for NF-κB activation and to examine the correlation with PIAS1 binding, six deletion mutants of N, each fused with a FLAG tag, were constructed; C11 (representing 1–112 aa), C51 (1–72 aa), C86 (1–37 aa), N18 (18–123 aa), N41 (41–123 aa), and N73 (73–123 aa). Then, NF-κB-luciferase assays were performed for each construct by co-transfecting with the NF-κB-luciferase reporter (Fig. 6b). The C11, C51, and N18 constructs showed the similar activity of NF-κB to that of full-length wild-type N (N-WT). N41 also activated NF-κB signaling but at a reduced rate than the above constructs. In contrast, C86 and N73 did not activate NF-κB, indicating the region between 37 and 72 aa of N was the essential domain for activation. Contained in this region are NLS and NoLS, suggesting that such localization signals might be crucial for NF-κB activation. To investigate the cellular distribution of N and its mutants, IFA was performed using anti-FLAG MAb. All mutants of N translocated to the nucleus including C86 and N73, although the NLS was absent in these mutants (Fig. 6c). We reasoned that C86 (37 aa) and N73 (50 aa) were small in their size and thus passively diffused to the nucleus as previously reported^65^. Nevertheless, the nucleolar localization of N appeared to be NoLS-dependent since C11, C51, N18, and N41 localized in the nucleolus (Fig. 6c), while C86 and N73 did not. This observation indicates the nuclear localization per se was not correlated with NF-κB activation in our transfection conditions. To determine the PIAS1-binding domain in N, we performed co-IP assays using individual deletion mutants. Cell lysates were precipitated using anti-HA MAb and probed with anti-FLAG PAb. N-WT, C11, N18, and N41 were pulled down by PIAS1 (Fig. 6d, second panel, lanes 2, 3, 6, 7), demonstrating that these constructs retained the PIAS1 binding ability. Since these constructs activated NF-κB (Fig. 6b), our results also indicate the positive correlation between PIAS1 binding of N and NF-κB activation. C51 was not initially detectable (Fig. 6d, second panel, lane 4), but with the increased amount of C51 plasmid for transfection and a longer exposure of the membrane, it was apparent that C51 was also pulled down by PIAS1 (Fig. 6d, third panel, lane 4), showing the binding of C51 to PIAS1. Taken altogether, these data indicate that the region between aa position 37 and 72 of N is the region for PIAS1 binding and also for NF-κB activation.

### NLS of N mediates NF-κB activation

**Figure 7:**
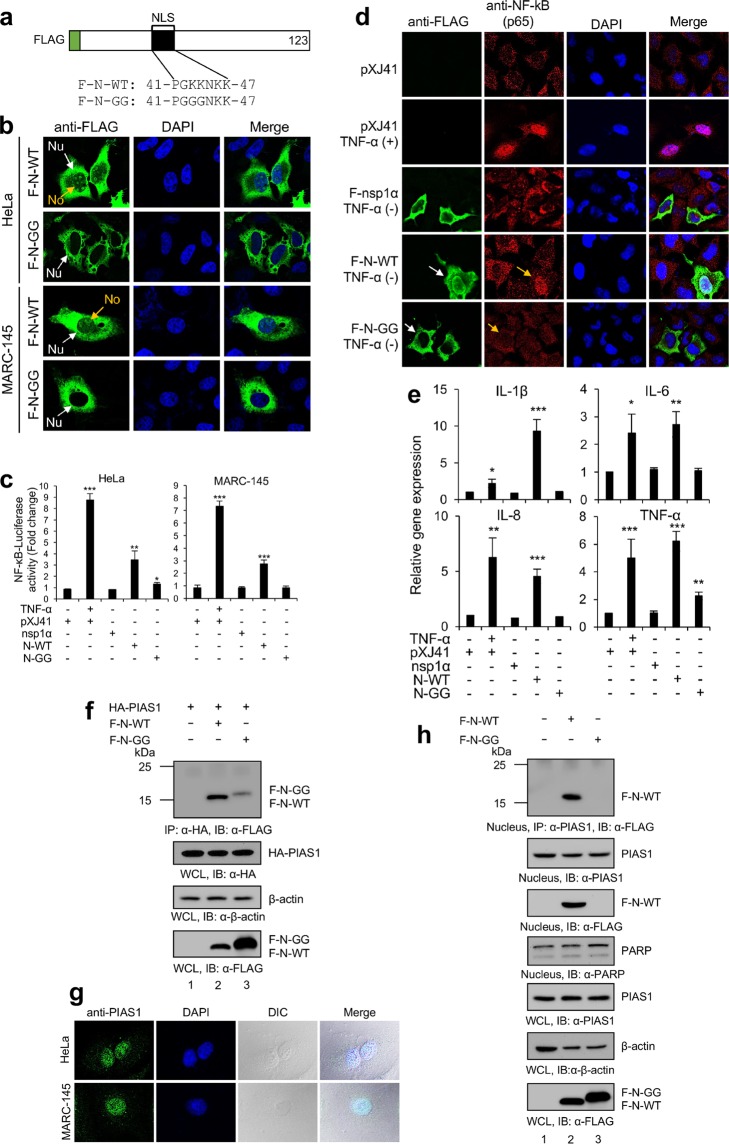
N-GG lost NF-κB activation and PIAS1 binding in the nucleus. (**a**) Construction of NLS knock-out N-GG mutant. (**b**) Cells were transfected with 2 μg of plasmids and stained with α-FLAG MAb (green). Nuclei were stained with DAPI (blue). nucleus (Nu), nucleolus (No). (**c**) Cells were transfected with pNF-κB-Luciferase (0.5 μg) and pRL-TK (0.05 μg), along with 0.5 μg of indicated genes. After 24 h (HeLa) or 42 h (MARC-145), cells were incubated with 20 ng/ml of TNF-α for 6 h. Cell lysates were subjected to luciferase assays. Reporter activities were calculated by normalizing to respective *Renilla* activities. Error bars, mean ± s.d. (n = 3). *P < 0.05, **P < 0.01, ***P < 0.001. (**d**) Cells were transfected for 24 h and treated with TNF-α (20 ng/ml) for 20 min. Cells were stained with antibodies as indicated. Nuclei (blue) were stained with DAPI. White arrows indicate N-expressing cells, and yellow arrow indicates p65 in the nucleus. **e**, Cells were transfected with 2 μg of indicated genes for 42 h and treated with 20 ng/ml of TNF-α for 6 h. Total cellular RNA was isolated and RT-qPCR was performed to measure the transcripts levels. Expression levels were calculated with 2^-ΔΔCT^ method by normalizing to that of β-actin. Error bars, mean ± s.d. (n = 3). *P < 0.05, **P < 0.01, ***P < 0.001. (**f**) HeLa cells were transfected with the 2 μg of indicated genes for 30 h and subjected to co-IP. Cell lysates were immunoprecipitated with α-HA MAb followed by probing with α-FLAG PAb or α-HA MAb (top panel). (**g**) HeLa or MARC-145 cells were grown on cover slips and stained with α-PAIS1 (green). DAPI represents the nucleus. DIC, differential interference contrast. (**h**) HeLa cells were transfected with either F-N-WT (2 μg) or F-N-GG (2 μg), and the nuclear complex co-IPs were performed as described in Materials and Methods.

The PIAS1 binding region (37 aa to 72 aa) of N includes NLS (41 aa to 47 aa) (Fig. 6d), and the previous studies have shown that NLS is a determinant for the PRRSV pathogenesis in pigs^45,46^. Thus, it was of an interest to examine the role of NLS in the N-mediated NF-κB activation. NLS functions by interacting with importin-α and importin-β via the conserved sequence of 41-PGKKNKK-47 in N^51^. We made an NLS knock-out N gene mutant to substitute two lysine (K) residues at 43 and 44 with two glycine (G) residues and designated this mutant as N-GG (Fig. 7a). By IFA, N-WT was distributed in the cytoplasm, nucleus, and nucleolar in both HeLa and MARC-145 cells whereas N-GG did not enter the nucleus nor the nucleolus of cells (Fig. 7b). When we examined the NF-κB activity using the luciferase assay, N-WT activated NF-κB in these cells; however, N-GG lost the activation drastically (Fig. 7c). In HeLa cells, the NF-κB activity induced by N-GG was only slightly higher than that of pXJ41 empty vector, but was largely reduced compared to N-WT. In MARC-145 cells, N-GG did not induce the NF-κB activity and there was no difference between N-GG and pXJ41. These results indicate that NLS of N contributes to the NF-κB activity. We further examined the cellular distribution of p65 in N-GG expressing cells (Fig. 7d). While N-WT caused the p65 translocation and accumulation in the nucleus, N-GG did not cause the p65 nuclear translocation and it was rather distributed in the cytoplasm, as with in the cells expressing nsp1α or empty vector, indicating no activation of NF-κB by N-GG. We also examined the proinflammatory cytokine expressions mediated by N-GG by RT-qPCR (Fig. 7e). TNF-α or N-WT promoted the expression of IL-1β, IL-6, IL-8, and TNF-α, but N-GG did not induce the production of IL-1β, IL-6, and IL-8. Interestingly, TNF-α expression was slightly increased by 2-fold compared to pXJ41, but it was only about one-third of the level by N-WT. Together, these results show that NLS of N was important for N-mediated proinflammatory cytokine productions which is likely via NF-κB activation.

### N-GG did not bind to PIAS1

Since N-WT activated NF-κB while N-GG lost this activity, the interaction of N-GG and PIAS1 was examined. We co-transfected the HA-PIAS1 and N-GG plasmids in the equal molar ratio and conducted a pull-down assay using anti-HA MAb to determine whether N-GG was able to bind PIAS1. Surprisingly, a considerably reduced amount of N-GG was pulled down by HA-PIAS1 (Fig. 7f, top panel, lane 3). N-GG binding to PIAS1 was not completely abolished when N-GG was over-expressed. Since PIAS1 resides in the nucleus and suppresses NF-κB^28^, we assumed that the lack of binding between N-GG and PIAS1 was likely due to their different cellular distributions. To examine this possibility, we performed PIAS1 staining. Regardless of cell types, PIAS1 was predominantly found in the nucleus (Fig. 7g), and this observation was consistent with the previous finding from mouse embryo fibroblasts (MEFs)^28^.

N-GG did not localize in the nucleus as expected and this was likely because NLS was destroyed (Fig. 7b). We also performed the nuclear complex co-IP. The nuclear lysates were prepared and subjected to co-IP using anti-PIAS1 MAb and to immunoblot using anti-FLAG PAb. Only N-WT was identified in the nuclear extract (Fig. 7h, third panel, lane 2), and N-GG was not detectable (third panel, lane 3). By co-IP, N-GG was not pulled down by PIAS1 (top panel, lane 3), indicating the absence of binding between N-GG and PIAS1 in the nucleus. The results showed that, although N-GG still retained a weak binding to PIAS1 when over-expressed, N-GG blocked the endogenous PIAS1-binding. Altogether, our data confirm the N-PIAS1 interaction leading to NF-κB activation.

### SUMOylation of N

**Figure 8:**
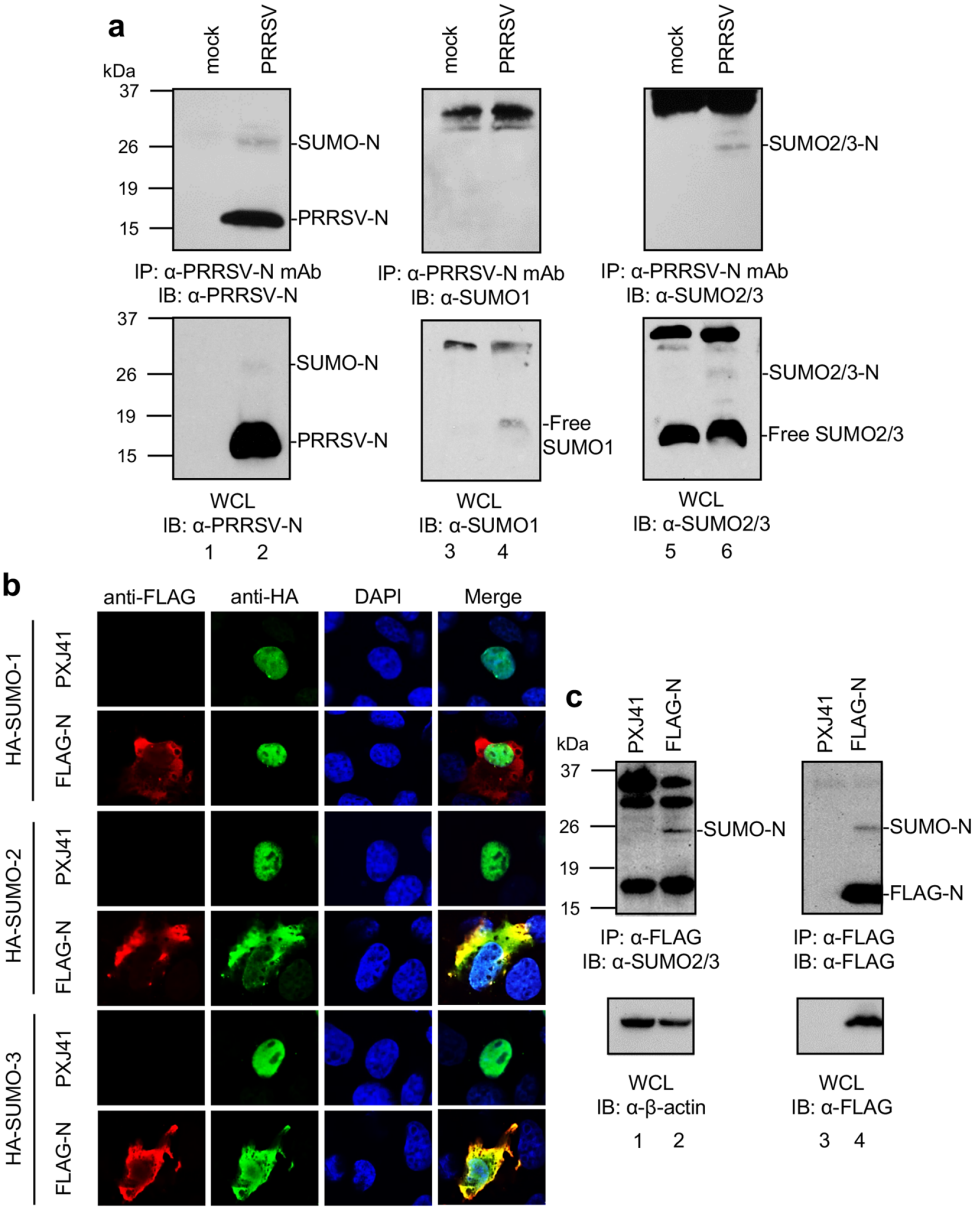
Modification of N by SUMO2/3 conjugation. (**a**) MARC-145 cells were infected with PRRSV at 5 moi for 48 h, and cell lysates were subjected to co-IP using α-PRRSV-N MAb (MR40) for immunoprecipitation and α-PRRSV-N PAb, α-SUMO1 MAb, or α-SUMO2/3 PAb for immunoblot (upper panels). Whole cell lysates (WCL) were subjected to Western blot as an input control (bottom panels). (**b**) HeLa cells were transfected with FLAG-N plasmid (2 μg) along with the HA-SUMO1 (2 μg), HA-SUMO2 (2 μg), or HA-SUMO3 (2 μg) plasmids. After 24 h, cells were stained with α-FLAG PAb (red) and α-HA MAb (green) to detect N and SUMO, respectively. Nuclei were stained with DAPI (blue). (**c**) HeLa cells were transfected with FLAG-N plasmid (2 μg) for 30 h, and cell lysates were subjected to co-IP using α-FLAG MAb for immunoprecipitation and α-SUMO2/3 PAb or α-FLAG PAb for immunoblot. Whole cell lysates (WCL) were subjected to Western blot as an input control (bottom panels), and β-actin served as a loading control.

PIAS1 is a SUMO E3-ligase^38^. Since N was shown to bind to PIAS1, it was of interest to examine if N would be a SUMO substrate of PIAS1. During the co-IP experiment using PRRSV-infected cells, we observed a 25 K protein in addition to N of 15 K (Fig. 5d, bottom panel, lane 2). The 25 K protein was routinely identified even under the denaturing conditions of SDS-PAGE, and its size was smaller than the dimer of N. The molecular weight of a SUMO molecule is 11 KDa, and thus we predicted that the 25 K protein was possibly a SUMOylated form of N^66,67^. To examine this possibility, cells were infected with PRRSV at 5 moi for 48 h and a co-IP assay was conducted using PRRSV-N MAb and SUMO-1 or SUMO-2/3 antibody. The 25 K protein was identified in the virus-infected cells (Fig. 8a, lane 2). This band was detectable by only SUMO-2/3 antibody in Western blot and co-IP (lane 6) but not by SUMO-1 antibody (lane 4). This finding indicates that N was conjugated by SUMO-2/3 modifier rather than by SUMO-1 modifier. To confirm this finding, IFA was performed using anti-HA Ab and anti-FLAG Ab in cells over-expressing HA-SUMO-1, HA-SUMO-2, or HA-SUMO-3 along with FLAG-N. It was evident that FLAG-N colocalized with HA-SUMO-2 or HA-SUMO-3 but did not colocalize with HA-SUMO-1 (Fig. 8b). SUMO-2/3 conjugated N protein was also detectable in FLAG-N expressing cells (Fig. 8c, upper panel, lanes 2 and 4). This finding was consistent with the report that the N protein of Chinese Highly Pathogenic (HP)-PRRSV was SUMOylated by interacting with the Ubc9^68^. Our data demonstrate that PRRSV N was SUMOylated by SUMO-2/3 modifier.

## Discussion

Proinflammatory cytokines play a wide range of cellular functions, and one major role is the contribution to host defense against infections. Production of proinflammatory cytokines is precisely balanced, and over-expression may cause tissue damages and immune-mediated diseases. PRRSV infects pulmonary alveolar macrophages (PAMs) in the lungs of pigs and causes inflammation and respiratory disease^5,6^. Expressions of IL-1β and TNF-α are driven by NF-κB, and in turn, they are potent NF-κB activators^22^. This positive feedback maintains the homeostasis of a host so that the host can quickly build an antiviral status during infection. IL-6 is induced by IL-1β and TNF-α^69^ and causes fever^70^. IL-8 is a neutrophil chemotactic factor and is also driven by NF-κB^71^. The production of such proinflammatory cytokines during PRRSV infection contributes to clinical and pathological outcome featured by fever, neutrophil infiltration to lungs, and tissue damages^10,12^. The roles of PRRSV in NF-κB regulation seem complicates since the suppression and the activation have both been reported. The suppression is largely executed by nonstructural proteins, including nsp1α, nsp1β, nsp4, nsp5, and nsp11^53–58^, whereas the activation is attributed to structural proteins of E and N (Fig. 2). The dual roles of PRRSV regulating NF-κB contribute to the overall pathogenesis of virus, but in cells and pigs, the virus as a whole activates NF-κB.

N is a potent viral activator for NF-κB (Fig. 2). Previous studies have shown that PRRSV activates NF-κB, and the N protein is the viral component inducing NF-κB activation^17–21,40,41^. However, the mechanism of action remains unknown, and in the present study, we have determined the molecular basis for N-mediated NF-κB activation. Proinflammatory cytokines including IL-1β, IL-6, IL-8, and TNF-α are upregulated and peak at 48 hpi in primary PAMs during infection (Fig. 1c). Our results show that N activates NF-κB luciferase signaling and induces the phosphorylation and nuclear translocation of p65 and confirms that N activates NF-κB (Figs 2 and 3). By yeast two-hybrid and co-IP assays, we have determined PIAS1 as an N-binding protein. PIAS1 is a known NF-κB repressor^28^, and we show that N-PIAS1 binding releases p65 and activates NF-κB.

Based on the results, we propose a working model for PRRSV-mediated NF-κB activation. In resting cells, PIAS1 functions to repress p65 in the nucleus to keep it in check, and therefore only minimal levels of cytokines are produced for homeostasis. At an early phase of infection, a limited amount of N is produced and the majority of N is used for progeny virus production in the cytoplasm. PRRSV N is a nuclear-cytoplasmic protein^43,44^, and thus some of N translocates to the nucleus, where it binds PIAS1 and releases p65. At this phase, most PIAS1 still remains as a complex with p65 and suppresses NF-κB, and the levels of proinflammatory cytokines are still low. As the infection proceeds, N concentration in the nucleus increases and the N binding to PIAS1 releases more p65. Released p65 then binds to the κB sites on DNA to initiate transcription of proinflammatory cytokine genes. Meanwhile, the replication process for viral genome will expose the viral RNA to the innate immune signaling cascade and activates relevant pathways which also triggers p65 phosphorylation in the cytoplasm. It has been shown that PRRSV infection induces the TLR4/MyD88 signaling pathway and NLRP3 inflammasome activation^13^. In addition, the expression of viral E protein contributes to the activation of inflammasome^21^. These factors together lead to the gradual expression of cytokines which are secreted from the cell and bind to their specific receptors to further stimulate the NF-κB signaling. As the N concentration becomes predominant in the nucleus, NF-κB activation is enhanced leading to inflammation.

Previously, we have constructed a series of N-NLS null-mutant PRRSVs and examined their pathogenesis in pigs^45,46^. These studies have demonstrated a correlation between the N nuclear localization and clinical attenuation of PRRSV. Since PIAS1 is predominantly nuclear^28^ and because N from NLS-null PRRSV remains in the cytoplasm, these studies support our model. Indeed, N-GG does not enter the nucleus and does not activate NF-κB (Fig. 7). Further studies show that losing NF-κB activity of N-GG is due to the lack of or reduced PIAS1 binding (Fig. 7). Since N neutralizes the repressive function of PIAS1, NF-κB singling is sensitized, and NF-κB activation becomes faster and synergistic especially when coinfection with other pathogens occurs.

Porcine respiratory disease complex (PRDC) is a multifactorial disease of pigs, and PRRSV is frequently reported as the major pathogen in the diseased animals. In pigs coinfected with PRRSV and *Mycoplasma hyopneumoniae* or other bacterial pathogens, the production of proinflammatory cytokines is elevated, and the clinical disease become severe^72–74^. Elevated production of proinflammatory cytokines by PRRSV will benefit the virus. Although inflammation is mounted during the late stage of infection, recruitments of monocytes and macrophages to infection site provide fresh target cells for virus^10^. Viruses take advantage by modulating the NF-κB signaling to facilitate their own good or to cause disease^1–3^. The modulatory actions may vary for different viruses^1–3^. PIAS1 is an important NF-κB repressor that controls inflammation, and the PIAS family proteins are involved in regulating immune-related gene expressions^34,35,75^. Despite some viral proteins have been reported to interact with the PIAS family proteins and are SUMOylated^66,67^, viral regulation of NF-κB through the PIAS family proteins is relatively new. VP35 of Ebola Zaire virus induces NF-κB activation in cells^76^ and interacts with PIAS1^77^. NP of influenza A virus also activates NF-κB^78^ and interacts with PIAS for SUMOylation^79^. The host Ubc9-mediated SUMOylation system was reported to exert adverse effects on PRRSV replication^68^. Since specific sites in N for SUMO conjugation are unknown, SUMOylation-negative mutant N proteins or mutant PRRSV cannot be generated for functional evaluation. Nevertheless, we report in this study that PRRSV N protein activates NF-κB through the mechanism of PIAS1 binding. The SUMOylation of N is an additional effect from this interaction, which further supports our finding of N-PIAS1 interaction. Our study presents a novel mechanism for PRRSV pathogenesis mediated by N and provides a new insight into viral regulation of inflammation by targeting the PIAS family proteins.

## Materials and Methods

### Cells and viruses

ZMAC macrophages were described elsewhere^52^, and kindly provided by Dr. F. Zuckermann (University of Illinois). HeLa (NIH AIDS Research and Reference Reagent Program, Germantown, MD) and MARC-145 cells were cultivated in Dulbecco’s modified Eagle’s medium (DMEM; Mediatech Inc., Manassas, VA), supplemented with 10% heat-inactivated fetal bovine serum (FBS; Gibco, Grand Island, NY), in a humidified incubator with 5% CO_2_ at 37 °C. Primary PAM cells were cultivated in RPMI-1640 medium (Mediatech Inc., Manassas, VA) supplemented with 10 mM glutamine and 10% heat-inactivated FBS. North American genotype PRRSV strain PA8 was propagated in MARC-145 cells and used for this study. For infection, cells were infected at a multiplicity of infection (moi) of 1 to 5 depending on experiments.

### Antibodies and chemicals

The antibodies and chemicals used in the present study are listed as follow. Anti (α)-PRRSV-N MAb (MR40) (mouse) was obtained from E. Nelson (South Dakota State University, Brookings, SD). α-p65 PAb (rabbit) (H-286, sc-7151), α-p65 MAb (mouse) (F-6, sc-8008), α-PIAS1 MAb (mouse) (F-1, sc-365127), α-PARP PAb (rabbit) (H-250, sc-7150), α-HSP90 MAb (mouse) (4F10, sc-69703), α-SUMO1 MAb (mouse) (D-11, sc-5308), α-SUMO2/3 PAb (rabbit) (FL-103, sc-32873), and α-β-actin MAb (mouse) (C4, sc-47778) were purchased from Santa Cruz Biotechnologies Inc. (Santa Cruz, CA). α-FLAG MAb (rat) (M2) was purchased from Agilent Technologies Inc. (Cedar Creek, TX). α-FLAG PAb (rabbit) was purchased from Rockland Inc. (Gilbertsville, PA). α-GFP MAb (rabbit), α-HA MAb (mouse), α-PIAS1 PAb (rabbit), and Alexa-Flour 488-conjugated and Alexa-Flour 568-conjugated secondary antibodies were purchased from ThermoFisher (Rockford, IL). α-HA MAb (rabbit) (C29F4), α-phospho-NF-κB p65 (Ser 536) MAb (rabbit) (93H1), and human Tumor Necrosis Factor-α (TNF-α) (8902) were purchased from Cell Signaling (Danvers, MA). The peroxidase-conjugated Affinipure goat anti-mouse IgG and peroxidase-conjugated Affinipure goat anti-rabbit IgG were purchased from Jackson Immuno Research (West Grove, PA). Lipopolysaccharides (LPS) and DAPI (4′, 6′-diamidino-2-phenylindol) were purchased from Sigma (St. Louis, MO).

### Genes and plasmids

The HA-PIAS1 and p65 genes were cloned into the pXJ41 expression vector. The nonstructural and structural genes were individually cloned in our laboratory from the FL12 infectious clone of PRRSV as the template^80^. The coding sequences were PCR-amplified to contain ATG translation initiation and TAG termination codons. A FLAG-tag was added at the N-terminus of each gene. For membrane protein genes such as nsp2, ORF2, ORF2a, ORF5, ORF5a, and ORF6, the FLAG-tag was added to the C-terminus. The C-terminal and N-terminal deletion mutants of PIAS1 gene (pHA-PIAS1-NTD and pHA-PIAS1-CTD, respectively) were cloned into pXJ41 using the Eco RI- and Xho I-recognition sequences. The series of N gene deletion mutant are described elsewhere^48^ and were subcloned to pXJ41. The pNF-κB-luciferase reporter plasmid was purchased from Stratagene Inc (La Jolla, CA). The pRL-TK *Renilla* luciferase reporter plasmid was purchased from Promega (Madison, WI). The pHA-SUMO1, pHA-SUMO2, and pHA-SUMO3 plasmids were obtained from Dr. L. Flamand (Laval University, Quebec, Canada).

### Immunofluorescence analysis (IFA)

HeLa or MARC-145 cells grown on microscope coverslips were fixed with 4% paraformaldehyde in PBS for 1 h at room temperature (RT), followed by three washes with PBS. Cells were permeabilized with 0.1% Triton X-100 for 15 min at RT, followed by three washes with PBS. After blocking with 1% BSA in PBS for 1 h at RT, cells were incubated with a primary antibody in blocking buffer for 2 h, followed by three washes with PBS and incubation with a secondary antibody for 1 h at RT. Cells were stained with DAPI in 1:5000 dilution for 5 min, and after a final wash with PBS, the coverslips were mounted on microscope slides using the Fluoromount-G mounting medium (Southern Biotech, Birmingham, AL). Cells were examined under the laser scanning confocal microscope (Nikon A1R).

### Reverse transcription-quantitative PCR (RT-qPCR)

RNA was extracted using the RNeasy mini kit according to the manufacturer’s instruction (QIAGEN, Hilden, Germany). RT-qPCR was performed in the ABI sequence detector system (ABI Prism 7000, Applied Biosystems, Life Technologies) using the final volume of 25 μl containing 3 μl of cDNA from reverse-transcription reaction, 12.5 μl of SYBR Green Master mix (Applied Biosystems), 2.5 μl of primer pairs (1.25 μl each of sense and antisense primers [10 μM]), and 7 μl of water. The primer sequences were designed as follow; for mIL-1β, forward 5′-CCCAACTGGTACATCAGCAC-3′, reverse 5′- GGAAGACACAAATTGCATGG-3′; for mIL-6, forward 5′-GCTGCAGGCACAGAACCA-3′, reverse 5′-AAAGCTGCGCAGGATGAGA-3′; for mIL-8, forward 5′- CTGGCGGTGGCTCTCTTG, 5′- CCTTGGCAAAACTGCACCTT-3′; for mTNF-α, forward, 5′-TCCTCAGCCTCTTCTCCTTCCT-3′, reverse 5′-ACTCCAAAGTGCAGCAGACAGA-3′; for mβ-actin, forward 5′-GCGCGGCTACAGCTTCACCAC-3′, reverse 5′-GGGCGCCAGGGCAGTAATCTC-3′; for pIL-1β, forward 5′- AACGTGCAGTCTATGGAGT-3′, reverse 5′-GAACACCACTTCTCTCTTCA-3′; for pIL-6, forward CTGGCAGAAAACAACCTGAACC-3′, reverse 5′- TGATTCTCATCAAGCAGGTCTCC-3′; for pIL-8, forward 5′-CCGTGTCAACATGACTTCCAA-3′, reverse 5′- GCCTCACAGAGAGCTGCAGAA-3′; pTNFα, forward 5′-AACCTCAGATAAGCCCGTCG-3′, reverse 5′- ACCACCAGCTGGTTGTCTTT-3′; for pβ-Actin, forward 5′- CTGGCATTGTCATGGACTCT-3′; reverse 5′-GCGATGATCTTGATCTTCAT-3′. The mRNA levels were calculated using the 2^−ΔΔCT^ method^81^ and normalized with respect to that of β-actin mRNA. Assays were repeated three times, and each assay was conducted in triplicate.

### DNA transfection and Dual luciferase reporter assay

DNA transfection was performed using Lipofectamine 2000 (Invitrogen, Carlsbad, CA) according to the manufacturer’s instructions. Cells were grown in 12-well plates, and in each well of the plate, 0.5 μg of pNF-κB-luciferase, 0.05 μg of pRL-TK, and 0.5 μg of the gene of interest were cotransfected. After 24 h (HeLa) or 42 h (MARC-145) post-transfection, cells were stimulated with TNF-α (20 ng/ml) for 6 h, and lysates were prepared using the passive lysis buffer (Promega). Supernatants were collected, and luciferase activities were determined using the Dual luciferase reporter assay system (Promega). Signals were obtained with the luminometer (Wallac 1420 VICTOR multi-label counter, Perkin Elmer, Waltham, MA). Values for firefly luciferase reporter activities were normalized using the *Renilla* internal control, and results were expressed as relative luciferase activities. The assay was repeated three times, each assay in triplicate.

### Co-immunoprecipitation and western blotting

Approximately one million cells of HeLa were transfected with plasmid DNA for 30 h. For virus infection, MARC-145 cells were infected with PRRSV at 5 moi for 48 h, and cell lysates were prepared in lysis buffer (50 mM Tris-HCl, 0.1% Triton-X-100, 150 mM NaCl, pH 7.8) containing 1X protease inhibitors (Thermo, Rockford, IL) by rotating at 4 °C for 30 min. Cell lysates were aliquoted to 100 μl as the whole cell lysates (WCL) and stored at −80 C until use. For the nuclear complex co-IP, the Nuclear Complex Co-IP kit (Active Motif, Carlsbad, CA) was used according to the manufacturer’s protocols. Cell lysates or nuclear extracts were incubated with antibody overnight at 4 °C. In the following morning, 30 μl of protein G agarose beads (EMD Millipore, Temecula, CA) were added and the mix was further incubated for at least 2 hr. Beads were washed three times with lysis buffer, and precipitates were eluted in 30 μl of M-PER mammalian protein extraction reagent (Thermo, Rockford, IL) and loading buffer by boiling at 95 °C for 5 min. The beads were spun down in a microcentrifuge and the supernatants were subjected to 10% SDS-PAGE and Western blot.

Small size proteins including PRRSV-N deletion mutants were resolved by gradient gel electrophoresis using Mini protein TGX 4–20% gel (Bio-Rad, Hercules, CA). Proteins were transferred to Immobilon-P PVDF membrane (Millipore), and the membranes were incubated in the TBST blocking buffer (10 mM Tris-HCl, 150 mM NaCl, 0.05% Tween-20 containing with 5% skim milk powder for 1 h at RT, followed by further incubation with primary antibody at 4 °C for overnight. The membranes were washed five times with TBST, and incubated with peroxidase-conjugated secondary antibody in TBST for another 1 h at RT. The membranes were washed five times, and the proteins were visualized using the ECL detection system (Thermo, Rockford, IL).

### Cell fractionations

HeLa cells were grown in 6-well-plates, and at 80% confluency, were transfected with 2 μg/well of plasmid DNA for 30 h. Cells were stimulated with TNF-α (20 ng/ml) for 20 min and fractionated using the Nuclear/Cytosol Fractionation kit (BioVision, Milpitas, CA) according to the manufacturer’s instructions with minor modifications. Briefly, cell monolayers were washed with PBS and collected in ice-cold PBS. Cells were centrifuged at 4 °C for 5 min at 1000 rpm in a microcentrifuge and resuspended in 200 μl of CEB-A (cytosolic extraction buffer-A) for incubation on ice for 10 min. Then, 11 μl of CEB-B were added to the lysates followed by vortex and incubation for 1 min on ice. The lysates were spun at 4 °C for 5 min in a microcentrifuge, and the supernatants were collected as the cytoplasmic fraction. The cell pellets were resuspended in the NEB buffer and incubated on ice for 10 min. This process of vortex-incubation was repeated 4 times. The nuclear pellet was centrifuged at 4 °C for 10 min in a microcentrifuge, and the supernatants were collected as the nuclear fraction. Both cytoplasmic and nuclear fractions were stored at −80 °C until use.

### Statistical analysis

Statistical significance was determined by two-tailed Student’s *t*-test with a value of *P* < 0.05 considered statistical significant.

## Supplementary information

Suplemmentary information

## References

[1] Rahman MM, McFadden G (2011). Modulation of NF-κB signalling by microbial pathogens. Nat Rev Microbiol.

[2] Santoro MG, Rossi A, Amici C (2003). NF‐κB and virus infection: who controls whom. EMBO J.

[3] Hiscott J, Kwon H, Genin P (2001). Hostile takeovers: viral appropriation of the NF-kappaB pathway. J Clin Invest.

[4] Lunney JK (2016). Porcine reproductive and respiratory syndrome virus (PRRSV): pathogenesis and interaction with the immune system. Annu Rev Anim Biosci.

[5] Albina E (1997). Epidemiology of porcine reproductive and respiratory syndrome (PRRS): an overview. Vet Microbiol.

[6] Loving CL, Brockmeier SL, Sacco RE (2007). Differential type I interferon activation and susceptibility of dendritic cell populations to porcine arterivirus. Immunology.

[7] Duan X, Nauwynck H, Pensaert M (1997). Effects of origin and state of differentiation and activation of monocytes/macrophages on their susceptibility to porcine reproductive and respiratory syndrome virus (PRRSV). Arch Virol.

[8] Liu Y (2010). Dynamic changes in inflammatory cytokines in pigs infected with highly pathogenic porcine reproductive and respiratory syndrome virus. Clin Vaccine Immunol.

[9] Miguel JC, Chen J, Van Alstine WG, Johnson RW (2010). Expression of inflammatory cytokines and Toll-like receptors in the brain and respiratory tract of pigs infected with porcine reproductive and respiratory syndrome virus. Vet Immunol Immunopathol.

[10] Van Reeth K, Labarque G, Nauwynck H, Pensaert M (1999). Differential production of proinflammatory cytokines in the pig lung during different respiratory virus infections: correlations with pathogenicity. Res Vet Sci..

[11] Van Reeth K, Nauwynck H (2000). Proinflammatory cytokines and viral respiratory disease in pigs. Vet Res.

[12] Liu J (2015). ICAM-1-dependent and ICAM-1-independent neutrophil lung infiltration by porcine reproductive and respiratory syndrome virus infection. Am J Physiol Lung Cell Mol Physiol.

[13] Bi J (2014). Porcine reproductive and respiratory syndrome virus induces IL-1beta production depending on TLR4/MyD88 pathway and NLRP3 inflammasome in primary porcine alveolar macrophages. Mediators Inflamm.

[14] Pineyro PE (2016). Modulation of Proinflammatory Cytokines in Monocyte-Derived Dendritic Cells by Porcine Reproductive and Respiratory Syndrome Virus Through Interaction with the Porcine Intercellular-Adhesion-Molecule-3-Grabbing Nonintegrin. Viral Immunol.

[15] Xiao Y (2015). The gene expression profile of porcine alveolar macrophages infected with a highly pathogenic porcine reproductive and respiratory syndrome virus indicates overstimulation of the innate immune system by the virus. Arch Virol.

[16] Lee YJ, Lee C (2012). Cytokine production in immortalized porcine alveolar macrophages infected with porcine reproductive and respiratory syndrome virus. Vet Immunol Immunopathol.

[17] Fu Y (2012). Porcine reproductive and respiratory syndrome virus induces interleukin-15 through the NF-kappaB signaling pathway. J Virol.

[18] Lee SM, Kleiboeker SB (2005). Porcine arterivirus activates the NF-kappaB pathway through IkappaB degradation. Virology.

[19] Song S (2013). Porcine reproductive and respiratory syndrome virus infection activates IL-10 production through NF-kappaB and p38 MAPK pathways in porcine alveolar macrophages. Dev Comp Immunol.

[20] Yu Z, Huang C, Zhang Q, Feng WH (2016). Porcine reproductive and respiratory syndrome virus (PRRSV) induces IL-12p40 production through JNK-AP-1 and NF-kappaB signaling pathways. Virus Res.

[21] Zhang K (2013). Porcine reproductive and respiratory syndrome virus activates inflammasomes of porcine alveolar macrophages via its small envelope protein E. Virology.

[22] Chen L-F, Greene WC (2004). Shaping the nuclear action of NF-kappaB. Nat Rev Mol Cell Biol.

[23] Dinarello CA (2000). Proinflammatory Cytokines. Chest.

[24] Saraiva M, O’Garra A (2010). The regulation of IL-10 production by immune cells. Nat Rev Immunol.

[25] Kagoya Y (2014). Positive feedback between NF-kappaB and TNF-alpha promotes leukemia-initiating cell capacity. J Clin Invest.

[26] Karin M, Ben-Neriah Y (2000). Phosphorylation meets ubiquitination: the control of NF-κB activity. Annu Rev Immunol.

[27] Lawrence T, Bebien M, Liu GY, Nizet V, Karin M (2005). IKK[alpha] limits macrophage NF-[kappa]B activation and contributes to the resolution of inflammation. Nature.

[28] Liu B (2005). Negative regulation of NF-κB signaling by PIAS1. Mol Cell Biol.

[29] Shuai K (2000). Modulation of STAT signaling by STAT-interacting proteins. Oncogene.

[30] Shuai K, Liu B (2003). Regulation of JAK–STAT signalling in the immune system. Nat Rev Immunol.

[31] Mohr S, Boswell R (1999). Zimp encodes a homologue of mouse Miz1 and PIAS3 and is an essential gene in Drosophila melanogaster. Gene.

[32] Betz A, Lampen N, Martinek S, Young MW, Darnell JE (2001). A Drosophila PIAS homologue negatively regulates stat92E. Proc Natl Acad Sci USA.

[33] Johnson ES, Gupta AA (2001). An E3-like factor that promotes SUMO conjugation to the yeast septins. Cell.

[34] Shuai K, Liu B (2005). Regulation of gene-activation pathways by PIAS proteins in the immune system. Nat Rev Immunol.

[35] Shuai K (2006). Regulation of cytokine signaling pathways by PIAS proteins. Cell Res.

[36] Chung CD (1997). Specific inhibition of Stat3 signal transduction by PIAS3. Science.

[37] Liu B (1998). Inhibition of Stat1-mediated gene activation by PIAS1. Proc Natl Acad Sci USA.

[38] Kahyo T, Nishida T, Yasuda H (2001). Involvement of PIAS1 in the sumoylation of tumor suppressor p53. Mol Cell.

[39] Liu B (2004). PIAS1 selectively inhibits interferon-inducible genes and is important in innate immunity. Nat Immunol.

[40] Luo R (2011). Activation of NF-kappaB by nucleocapsid protein of the porcine reproductive and respiratory syndrome virus. Virus Genes.

[41] Wongyanin P (2012). Role of porcine reproductive and respiratory syndrome virus nucleocapsid protein in induction of interleukin-10 and regulatory T-lymphocytes (Treg). J Gen Virol.

[42] Snijder EJ, Kikkert M, Fang Y (2013). Arterivirus molecular biology and pathogenesis. J Gen Virol.

[43] Rowland R, Kervin R, Kuckleburg C, Sperlich A, Benfield DA (1999). The localization of porcine reproductive and respiratory syndrome virus nucleocapsid protein to the nucleolus of infected cells and identification of a potential nucleolar localization signal sequence. Virus Res.

[44] Rowland RR (2003). Peptide domains involved in the localization of the porcine reproductive and respiratory syndrome virus nucleocapsid protein to the nucleolus. Virology.

[45] Lee C (2006). Mutations within the nuclear localization signal of the porcine reproductive and respiratory syndrome virus nucleocapsid protein attenuate virus replication. Virology.

[46] Pei Y (2008). Functional mapping of the porcine reproductive and respiratory syndrome virus capsid protein nuclear localization signal and its pathogenic association. Virus Res.

[47] Yoo D, Wootton SK, Li G, Song C, Rowland RR (2003). Colocalization and interaction of the porcine arterivirus nucleocapsid protein with the small nucleolar RNA-associated protein fibrillarin. J Virol.

[48] Song C, Lu R, Bienzle D, Liu HC, Yoo D (2009). Interaction of the porcine reproductive and respiratory syndrome virus nucleocapsid protein with the inhibitor of MyoD family-a domain-containing protein. Biol Chem.

[49] Yoo D (2010). Modulation of host cell responses and evasion strategies for porcine reproductive and respiratory syndrome virus. Virus Res.

[50] Wang X (2012). Poly(A)-binding protein interacts with the nucleocapsid protein of porcine reproductive and respiratory syndrome virus and participates in viral replication. Antivir Res.

[51] Rowland RR, Yoo D (2003). Nucleolar-cytoplasmic shuttling of PRRSV nucleocapsid protein: a simple case of molecular mimicry or the complex regulation by nuclear import, nucleolar localization and nuclear export signal sequences. Virus Res.

[52] Chen W-Y, Schniztlein WM, Calzada-Nova G, Zuckermann FA (2018). Genotype 2 strains of porcine reproductive and respiratory syndrome virus dysregulate alveolar macrophage cytokine production via the unfolded protein response. J Virol.

[53] Chen Z (2010). Identification of two auto-cleavage products of nonstructural protein 1 (nsp1) in porcine reproductive and respiratory syndrome virus infected cells: nsp1 function as interferon antagonist. Virology.

[54] Jing H (2017). Porcine reproductive and respiratory syndrome virus nsp1α inhibits NF-κB activation by targeting the linear ubiquitin chain assembly complex. J Virol.

[55] Subramaniam S (2010). Porcine reproductive and respiratory syndrome virus non-structural protein 1 suppresses tumor necrosis factor-alpha promoter activation by inhibiting NF-kappaB and Sp1. Virology.

[56] Song C, Krell P, Yoo D (2010). Nonstructural protein 1α subunit-based inhibition of NF-κB activation and suppression of interferon-β production by porcine reproductive and respiratory syndrome virus. Virology.

[57] Huang C (2014). Porcine reproductive and respiratory syndrome virus nonstructural protein 4 antagonizes beta interferon expression by targeting the NF-κB essential modulator. J Virol.

[58] Sun Y (2016). Nonstructural protein 11 of porcine reproductive and respiratory syndrome virus suppresses both MAVS and RIG-I expression as one of the mechanisms to antagonize type I interferon production. PLoS One.

[59] Fang Y (2012). Porcine reproductive and respiratory syndrome virus nonstructural protein 2 contributes to NF-κB activation. Virol J.

[60] Sun Z, Chen Z, Lawson SR, Fang Y (2010). The cysteine protease domain of porcine reproductive and respiratory syndrome virus nonstructural protein 2 possesses deubiquitinating and interferon antagonism functions. J Virol.

[61] Kim H, Kwang J, Yoon I, Joo H, Frey M (1993). Enhanced replication of porcine reproductive and respiratory syndrome (PRRS) virus in a homogeneous subpopulation of MA-104 cell line. Arch Virol.

[62] Weiskirchen R (2001). LIM-domain protein cysteine-and glycine-rich protein 2 (CRP2) is a novel marker of hepatic stellate cells and binding partner of the protein inhibitor of activated STAT1. Biochem J.

[63] Wootton SK, Yoo D (2003). Homo-oligomerization of the porcine reproductive and respiratory syndrome virus nucleocapsid protein and the role of disulfide linkages. J Virol.

[64] Doan DN, Dokland T (2003). Structure of the nucleocapsid protein of porcine reproductive and respiratory syndrome virus. Structure.

[65] Timney, B. L. *et al*. Simple rules for passive diffusion through the nuclear pore complex. *J Cell Biol*, 201601004 (2016). 10.1083/jcb.201601004 PMC5057280 27697925

[66] Everett RD, Boutell C, Hale BG (2013). Interplay between viruses and host sumoylation pathways. Nat Rev Microbiol.

[67] Wimmer P, Schreiner S, Dobner T (2012). Human pathogens and the host cell SUMOylation system. J Virol.

[68] Wang C (2017). Interaction of porcine reproductive and respiratory syndrome virus proteins with SUMO-conjugating enzyme reveals the SUMOylation of nucleocapsid protein. PLoS one.

[69] Zoja C (1991). Interleukin-1 β and Tumor Necrosis Factor-α Induce Gene Expression and Production of Leukocyte Chemotactic Factors, Colony-stimulating Factors, and Interleukin-6 in Human Mesangial Cells. Am J Pathol.

[70] Fossum C, Wattrang E, Fuxler L, Jensen KT, Wallgren P (1998). Evaluation of various cytokines (IL-6, IFN-α, IFN-γ, TNF-α) as markers for acute bacterial infection in swine-a possible role for serum interleukin-6. Vet Immunol Immunopathol.

[71] Lin G (1994). Regulation of interleukin-8 expression in porcine alveolar macrophages by bacterial lipopolysaccharide. J Biol Chem.

[72] Thanawongnuwech R, Thacker B, Halbur P, Thacker EL (2004). Increased production of proinflammatory cytokines following infection with porcine reproductive and respiratory syndrome virus and Mycoplasma hyopneumoniae. Clin Diagn Lab Immunol.

[73] Qiao S (2011). Porcine reproductive and respiratory syndrome virus and bacterial endotoxin act in synergy to amplify the inflammatory response of infected macrophages. Vet Microbiol.

[74] Thanawongnuwech R, Young TF, Thacker BJ, Thacker EL (2001). Differential production of proinflammatory cytokines: *in vitro* PRRSV and Mycoplasma hyopneumoniae co-infection model. Vet Immunol Immunopathol.

[75] Liu B, Shuai K (2008). Targeting the PIAS1 SUMO ligase pathway to control inflammation. Trends Pharmacol Sci.

[76] Gantke T (2013). Ebola virus VP35 induces high-level production of recombinant TPL-2-ABIN-2-NF-kappaB1 p105 complex in co-transfected HEK-293 cells. Biochem J.

[77] Chang TH (2009). Ebola Zaire virus blocks type I interferon production by exploiting the host SUMO modification machinery. PLoS Pathog.

[78] Flory E (2000). Influenza virus-induced NF-κB-dependent gene expression is mediated by overexpression of viral proteins and involves oxidative radicals and activation of IκB kinase. J Biol Chem.

[79] Han Q (2014). Sumoylation of influenza A virus nucleoprotein is essential for intracellular trafficking and virus growth. J Virol.

[80] Truong HM (2004). A highly pathogenic porcine reproductive and respiratory syndrome virus generated from an infectious cDNA clone retains the *in vivo* virulence and transmissibility properties of the parental virus. Virology.

[81] Livak, K. J. & Schmittgen, T. D. Analysis of relative gene expression data using real-time quantitative PCR and the 2-DDCT method. *Methods* **25** (2001). 10.1006/meth.2001.1262 11846609

