# Supplementary material for: Interaction of PIAS1 with PRRS virus nucleocapsid protein mediates NF-κB activation and triggers proinflammatory mediators during viral infection
Source: Sci Rep. 2019 Jul 30;9:11042. doi: 10.1038/s41598-019-47495-9 (PMC6667501; doi:10.1038/s41598-019-47495-9)
Supplement: Supplementary file 1 — Suplemmentary information [file 41598_2019_47495_MOESM1_ESM.pdf]

Interaction of PIAS1 with PRRS virus nucleocapsid protein mediates NF-κB activation and triggers proinflammatory mediators during viral infection

Hanzhong Ke [1], Sera Lee [1], Jineui Kim [1], Hsiao-Ching Liu [2], Dongwan Yoo [1]*

[1] Department of Pathobiology, University of Illinois at Urbana-Champaign, Urbana, IL, USA.

[2] Department of Animal Science, North Carolina State University, Raleigh, NC, USA.

*Address correspondence to:

Dr. Dongwan Yoo

Department of Pathobiology, University of Illinois at Urbana-Champaign,

2001 South Lincoln Ave, Urbana, IL, 61802

Email: dyoo@illinois.edu

Phone: 217-244-9120

Word count: Abstract: 195 words; Text: 5412 words.

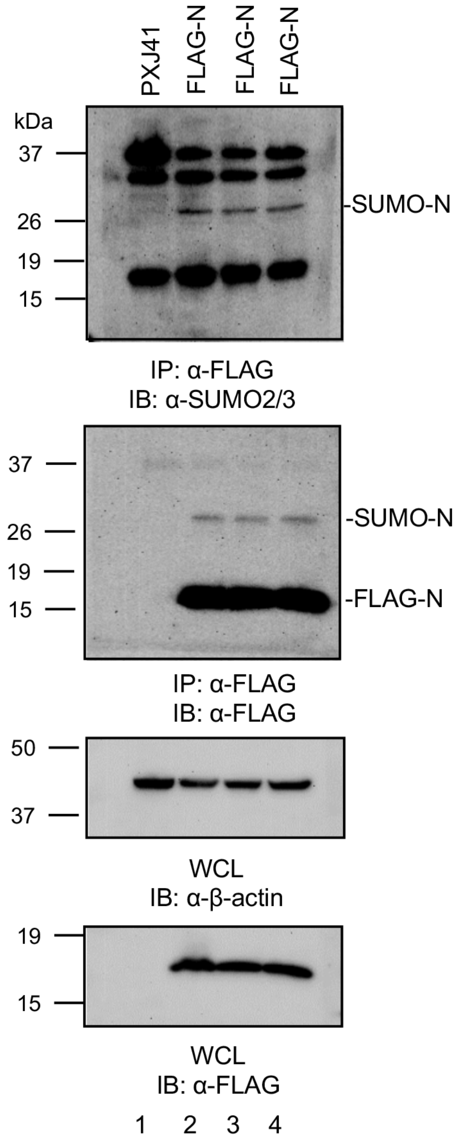

Supplementary figure S1. Modification of N by SUMO2/3 conjugation. HeLa cells were transfected with FLAG-N plasmid (2 μg) for 30 h (in triplicate), and cell lysates were subjected to co-IP using α-FLAG MAb for immunoprecipitation and α-SUMO2/3 PAb or α-FLAG PAb for immunoblot. Whole cell lysates (WCL) were subjected to Western blot as an input control (bottom panels), and β-actin served as a loading control. Right two lanes (lanes 3 & 4) were duplicates of lane 2 thus were cropped as presented in Fig. 8**c**.
